# Surf Session Events’ Profiling Using Smartphones’ Embedded Sensors †

**DOI:** 10.3390/s19143138

**Published:** 2019-07-17

**Authors:** Diana Gomes, Dinis Moreira, João Costa, Ricardo Graça, João Madureira

**Affiliations:** 1 Fraunhofer Portugal AICOS, 4200-135 Porto, Portugal; 2 Faculty of Engineering, University of Porto, 4200-465 Porto, Portugal

**Keywords:** activity recognition, gps, inertial sensors, monitoring system, smartphone, sports performance, surf

## Abstract

The increasing popularity of water sports—surfing, in particular—has been raising attention to its yet immature technology market. While several available solutions aim to characterise surf session events, this can still be considered an open issue, due to the low performance, unavailability, obtrusiveness and/or lack of validation of existing systems. In this work, we propose a novel method for wave, paddle, sprint paddle, dive, lay, and sit events detection in the context of a surf session, which enables its entire profiling with 88.1% accuracy for the combined detection of all events. In particular, waves, the most important surf event, were detected with second precision with an accuracy of 90.3%. When measuring the number of missed and misdetected wave events, out of the entire universe of 327 annotated waves, wave detection performance achieved 97.5% precision and 94.2% recall. These findings verify the precision, validity and thoroughness of the proposed solution in constituting a complete surf session profiling system, suitable for real-time implementation and with market potential.

## 1. Introduction

Surfing is a popular sport all over the world and can be practiced in both leisurely or competitive ways. Minimal training and equipment makes this an appealing water sport for people to experiment and enjoy. Surfing consists in riding a surfboard along the unbroken section (or wall) of a wave, as it travels towards the shore. The main piece of equipment required to perform this sport is a foam and fibreglass surfboard, where the surf practitioner is expected to stand up erect on his feet in a wave ride [1].

Lately, the market demand for surfboards has shown an increase in volume, mainly for advanced level surf practitioners. This market is expected to grow 12.24% during 2018–2022 [2]. Due to its increasing popularity worldwide, new surf-related solutions have been developed in order to assist surfers in catching the best waves. For example, surf-specific websites such as *MagicSeaWeed* [3] or *Windguru* [4] provide weather forecast and sea conditions, constituting an essential tool for passionate surfers. Following this trend, the analysis and extraction of performance-related measurements to create automatic and accurate surf session profiles shall constitute an appealing and challenging research area with high commercial potential [1], as there is, indeed, limited available information for surf practitioners and coaches in terms of key performance analytics that are currently quite commonly present for other sports [1,5]. Most of these sports monitoring systems generate biofeedback based on sensor data retrieved during the training session [5,6,7], and are capable of providing useful information about the executed movements, as they are correctly or incorrectly performed [6,7]. Moreover, in surfing, as in most sports, evolving towards more sophisticated movements or manoeuvres requires the mentoring of a more experienced person, such as an instructor or coach; sensorisation and movement quantification can also assist this process.

A surf session is usually constituted by four main events: paddle, stationary, wave riding and miscellaneous events (i.e., events such as wading or duck diving that seldom occur) [8]. Wave riding is the most important activity; however, it only typifies 3.8% of the surf session’s total time. Miscellaneous events have a low impact in the overall session, representing only 2.2%. The major events are paddling, accounting for 51–54% of the total session time, and stationary events, such as sitting or laying on the board (42.5%) [9]. This distribution presents its own challenges in terms of developing surf monitoring solutions, due to the scarcity of some activities; in particular, wave riding, the most important surfing event, is very rare, increasing the difficulty to accurately annotate and monitor it.

Currently, most solutions base their estimations of speed, distance and movement patterns on Global Positioning System (GPS) measurements [10,11,12,13], using them to evaluate the performance of the surfer [5,14]. However, given the nature of this sport, and especially during manoeuvres, short and very intense periods of activity may be inaccurately characterised when solely using GPS data, making the estimated surf-related metrics unreliable [5,14,15]. Moreover, stronger accelerations have been proven to negatively impact instantaneous velocity measurements provided by GPS data alone, i.e., higher accelerations frequently compromise the GPS’ speed estimation [5,15]. This is extremely relevant in the context of a surf session, since these solutions may not be suitable for computing performance-related metrics with the validity and reliability that surf practitioners need. These limitations could be mitigated by combining the use of GPS with a secondary source of data, namely inertial sensors [6,7,10,16,17,18]. Indeed, Chambers et al. [6] stated that, in sport performance analytics, speed and distance calculations are frequently performed using GPS, but sport-specific movements are identified and characterised using inertial sensors. These sensors are widely spread and increasing its popularity, especially for biomedical and sports applications [7,19,20], due to its small size, low cost, low energy requirements and the fact that they are embedded in many ubiquitous devices. Inertial measurement units (IMU) are usually composed by a triaxial accelerometer, gyroscope and magnetometer, measuring acceleration, angular velocity and magnetic field intensity, respectively, and are generally used to estimate position and orientation by tracking rotational and translational movements [7,16]. Several strategies have been proposed in recent years to overcome the most common inertial measurement-related errors (e.g., drift), usually based on sensor fusion methods which try to compensate for each sensor’s well-known sources of error by employing sophisticated filtering techniques, which ultimately output reliable orientation estimations [16,20].

This context motivated the development of a new algorithm for the detection and characterisation of surf session events from intermediate to advanced level surfers, combining both IMU and GPS data sources to achieve the most reliable outcome possible. In this sense, this manuscript presents an extension of the work reported in [17], with an extended validation of the entire surf session profiling algorithm, namely concerning wave detection performance, with an improved dataset comprising approximately 7.5 h of annotated data and featuring a total of 327 waves, and an additional contribution:

• Introduction of a novel and improved module of laying events classification, focusing in the distinction of paddle, sprint paddle, dives, and idle laying, using frequency-domain features.

The remainder of the paper is organised as follows. Section 2 describes the prior work conducted in this field. Section 3 describes all necessary equipment and methodology, which made this study possible. Section 4 and Section 5 report and discuss the main findings, respectively. Finally, Section 6 highlights the main conclusions of this study and points out possible directions for future work.

## 2. Related Work

There are a few commercially available solutions for surf monitoring. *Rip Curl Search GPS* watch [11] uses GPS signals to perform some estimations and deliver a few surf-related metrics, such as wave count, travelled distance and wave speed. Solutions which mostly rely on GPS data alone to perform their core estimations are hardly able to detect complex surfing movements, such as in-wave manoeuvres, and may lack precision in the determination of the boundaries of some events (e.g., take-off and end-ride moments). Some mobile applications have also been developed (e.g., *Surf Track* [21], *Dawn Patrol* [22], and *WavesTracker* [23]), but very little information is provided regarding their functioning, performance and system setup.

*Glassy Pro* is a wristband which combines GPS and inertial sensor data, namely the accelerometer and gyroscope sensors, in order to improve detection and characterisation of surf-related events [12]. GPS data are mostly used for wave detection while inertial sensors are used to explore and detect other events, such as paddling periods. The fact that it is intended to be a wrist-worn device has both advantages and disadvantages. On the one hand, it is practical and mostly unobtrusive. On the other hand, monitoring surf movements based on wrist motion may be insufficient to retrieve the most useful information in some cases, especially during wave rides, in particular if one intends to monitor rotations. Torso and board rotation are important metrics for wave performance analysis and these will most likely be lost if wrist movement is the only one under analysis, due to the variety of movements of great amplitude enabled by the shoulder joint.

*Trace Up* is a dedicated device designed to be placed in the nose of the surfboard. It uses GPS and inertial sensors (gyroscope and magnetometer) to identify and analyse complex surf movements during a session [13]. By combining several sources of data and their strategic placement on the board, it can provide information about wave riding times, distance, speed, number of turns and turn angle values. While this device provides a handful of useful features for surf monitoring, no performance evaluation studies were found to validate its detections and measurements. In addition, to the best of the authors’ knowledge, this product is no longer available for tracking surf sessions, since the scope of the company has evolved for focusing solely in football tracking [13]. *Xensr Air* is another product, very similar to the previous [24]; the device was also designed to be mounted in the surfboard, and uses GPS and a sensor fusion approach to monitor and extract surf session metrics; however, to best of the authors’ knowledge, this product is currently unavailable for purchase [24].

Besides commercially available solutions, some research studies also aim at surf monitoring and/or performance analysis. Madureira et al. [10] proposed an algorithm for wave detection which compared the use of GPS sensor alone against its combination with inertial sensors data, placing a smartphone on the upper back of the surfer. The results indicate that wave detection is more accurate when the algorithm uses both data sources (inertial and positioning sensors). This finding is consistent with that of our previous work [17], which tested this premise under more demanding circumstances and with a significantly bigger dataset. The addition of inertial sensors also improved their definition of wave boundaries. Moreover, false positives were more frequent when using only speed estimation from GPS as a feature [10].

In another study, Hoettinger et al. [25] proposed a machine learning approach for activity recognition in surf sessions using inertial data from a device placed on the surfer’s chest, inside a waterproof case, under the wetsuit. The goal of this work was to correctly identify specific events of a surf session, namely differentiating between wave and non-wave events, using two different machine learning techniques. The results show that both techniques could be suitable to fulfil this purpose, with only seven waves incorrectly classified from a total of 214 annotated waves [25].

## 3. Material and Methods

### 3.1. Data Collection and Annotation

The first and one of the most challenging steps within this study was related to the data collection process. For this purpose, two Android applications and a video camera were used. The video camera was placed on the beach at the spot which enabled the clearest view of the surfing area to record the whole surf session, constituting the ground truth of all validated events in this study. A simple Android data collection application was created to record data from the inertial sensors—accelerometer, gyroscope and magnetometer—and GPS, sampled at 100 Hz and 1 Hz, respectively. Another Android application was created and used to assist a real-time annotation of relevant events, by featuring three buttons, which defined the following moments: in/out of water, paddling and wave riding periods. This application was handled by a third person (usually a researcher) providing assistance to the real-time data annotation from the beach. This process was planned to facilitate the posterior offline annotation, especially in cases when the surfer cannot be spotted on camera while performing a relevant activity. For each new data collection, both Android applications were initiated at the same time, with the start moment (when the *Start* button is pressed) recorded by the video camera. This protocol enabled posterior data synchronisation between all recording devices.

**Figure 1:**
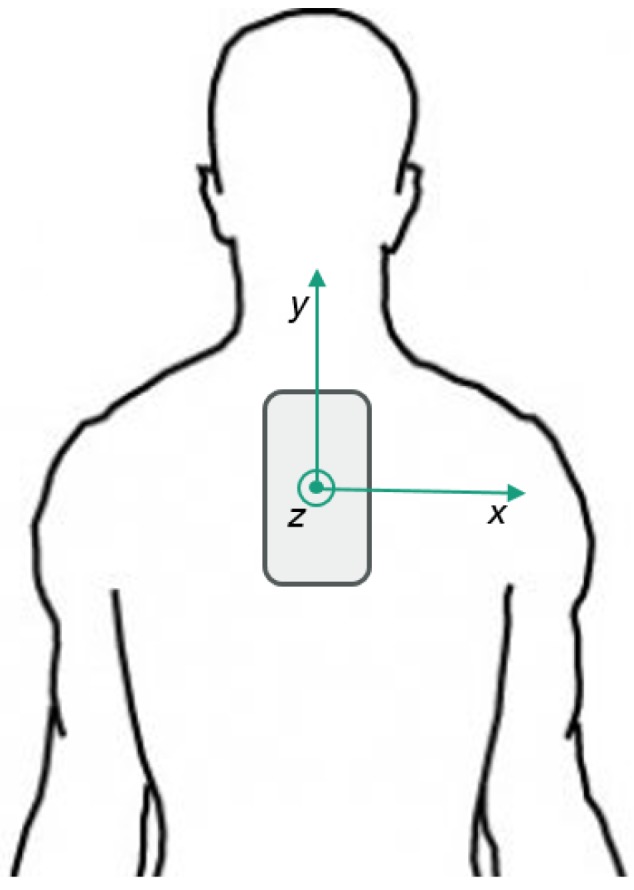
Smartphone positioning in the surfer’s upper torso, with sensor frame’s representation.

In every data collection session, a *Samsung Galaxy A3 (2017)* smartphone, with the data recording application installed, was placed vertically between the shoulder blades of the volunteer surfer, inside a waterproof case under the wetsuit. Figure 1 illustrates the positioning and configuration of the smartphone device.

Especially in surf, capturing data using sensors can be challenging, as surfers are often not too keen on using electronic devices at sea due to its inherent risks and/or the fact that device’s positioning can be uncomfortable, affecting performance itself [10]. Thus, the proposed smartphone positioning was derived from a set of 15 interviews conducted with experienced surfers. This site was considered the most adequate on-body location to place the device since it should not particularly disturb surf-related movements. Using a smartwatch as the sensing device was also considered; however, during the conducted interviews, some surfers also referred that they were not fond and did not use these devices because they hinder paddling movements. Moreover, placing the sensing device in the upper torso would enable the tracking of in-wave rotations and torsion, which can hardly be accurately monitored based on wrist sensorisation due to the amplitude of movements enabled by the wrist–elbow–shoulder joints.

Thirteen different surfers participated voluntarily in a combined total of 17 data collection sessions, after giving their explicit informed consent for the collection of all required data, including video. The recruited surfers had different background and expertise, ranging from intermediate to advanced levels. All sessions were annotated with frame precision using Kinovea software [26], with the recorded videos used as the ground truth, and cross-checked with the real-time annotations. In the ideal scenario, all surf related events should be annotated; however, not all videos were equally appropriate in maintaining a clear view of the surfers’ activities, for example due to sea waves and agitation, which can obstruct their direct observation. This is quite frequent in sessions that occurred with rough sea or bad weather conditions, with implications in the annotation of the events of interest. Therefore, we opted for only annotating periods in which the annotator was completely certain about the on-going event. Fortunately, this issue had less impact in the annotation of waves, since riding a wave implies sliding its wall, usually at a greater height than the occlusion causers. Some of the data collections also took place with two participants at once, while using only one video camera, which limited the total on-camera time of each surfer. Moments when the surfer was off camera sight were not annotated, and, therefore, not considered for comparison in the validation stage.

Given the importance of not missing any wave period, the annotation application was used to assist the process of wave annotation, not only by making this process faster and effective but also by guaranteeing that, even if, for some reason, a wave period were not recorded on camera, it would not be totally lost, even though its limits were annotated with less precision. Table 1 describes the two datasets in which data were split with respect to annotation process. The *Waves dataset* was only annotated for wave riding periods from all collected surf sessions, while the *All events dataset* is composed by a smaller subset of surf sessions that were annotated for all surf events, including paddling (sprint and regular), stationary moments, duck diving and wave riding periods.

### 3.2. Events Detection

Three of the sessions were recorded with a previous version of the data collection application sampling inertial sensors’ data at 50 Hz, instead of 100 Hz. This sampling frequency was found to be enough to be used by the proposed algorithm; therefore, data from all the remaining sessions were under-sampled by a factor of 2 (to 50 Hz).

**Figure 2:**
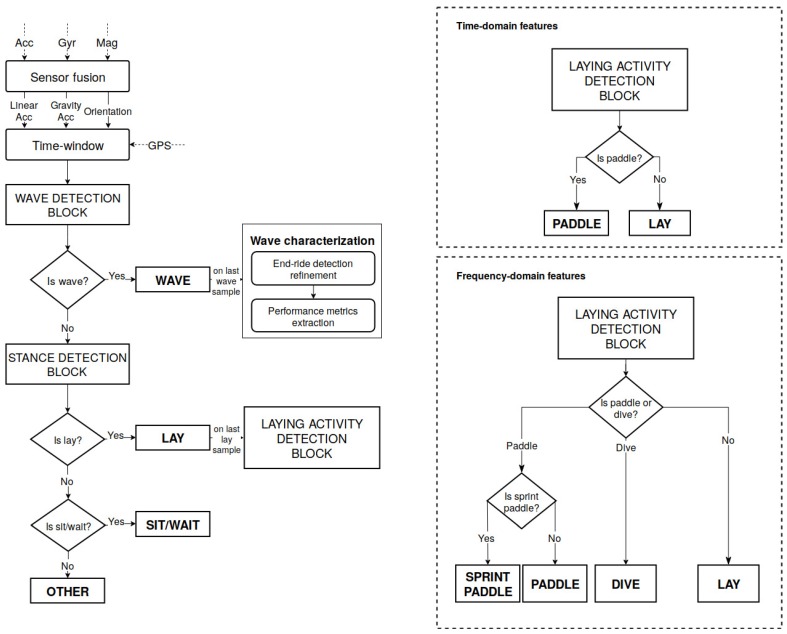
Flow of operations of the core event-detection algorithm, evidencing the two different implementations of the laying activity detection block.

A sensor fusion approach based on the combination of accelerometer, gyroscope and magnetometer sensor readings was used to overcome several individual sensor limitations. The gradient descent based orientation filter authored by Madgwick et al. [16] was used to fulfil this purpose. This approach has been widely employed and recognised among the research community and is frequently used in low processing power devices, associated with accurate results [27]. The use of a sensor fusion strategy based on gravity and magnetic field directions enabled the creation of a relative local frame of reference, the North–East–Down (NED) frame, with the *Down* axis being defined by the measured gravity direction provided by the accelerometer; the *North* axis being defined by the measured Earth magnetic field direction provided by the magnetometer; and the *East* axis being defined as perpendicular to the previous, following the right hand rule. This orientation filter uses quaternions for storing and representing orientation changes, and accelerometer and magnetometer data in an analytically derived and optimised gradient descent algorithm to compute the direction of the gyroscope measurement error as a quaternion derivative [16]. The filter output quaternion represents an optimised and more robust estimation of the actual orientation. Unlike Euler angles representation, quaternions do not suffer from gimbal lock and can be used to express an orientation/rotation both in the relative NED frame or in the device’s own inertial frame. This sensor fusion strategy enabled a more accurate dissociation between linear and gravitational components of the accelerometer readings, by using a better estimation of device’s current orientation. Linear acceleration components were estimated by projecting the NED frame fixed gravity vector onto the device’s own frame and then subtracting it from the current accelerometer reading. Gravity, linear acceleration and orientation signals constituted the basis for the detection of all surf-related events, as Figure 2 illustrates.

The data stream segmentation strategy took into account that the complex activities under analysis usually take several seconds; thus, windows of length in the order of seconds should be a proper fit for the problem in hands. Moreover, near real-time response was demanded. As such, the proposed algorithm splits the data stream into fixed-length windows of 1 s with 50% overlap, i.e., the algorithm returns a new activity prediction every 0.5 s, based on the samples from the previous second. Each of these windows was processed until a decision was obtained, being the respective label assigned. Thus, a decision on the activity that is currently being performed is obtained every half second. However, events were only processed after the detection of their end, so that post-processing verifications could be performed. The following subsections detail the necessary steps and processing involved in each block for detecting these activities, in accordance with the flow depicted in Figure 2.

#### 3.2.1. Waves

In [17], Gomes et al. investigated the pros and cons of using different sensors for wave detection, finally concluding that the combination of both GPS and IMU sensor data was the overall better approach. This approach was, therefore, selected for the subsequent work reflected in this manuscript.

As such, wave detection was based on the establishment of intelligible criteria, directly derived from the observation of wave events. These criteria were sequentially implemented as rules which define a wave period (if verified), and can be summarised as follows:

- Take-off can only occur following a laying stance or a transition period (class *other*).
- Take-off involves a fast increase of the surfer’s speed.
- Wave riding implies maintaining high speed and strong acceleration.

These criteria were verified by implementing a threshold-based approach taking acceleration and speed derived features as input, computed from each new time-window arriving to the wave detection block. Surfers’ in-wave acceleration status was estimated based on the magnitude of linear acceleration in *Y* and *Z* axes in the sensor frame; translation speed was estimated based on GPS measurements. Thresholds were mostly empirically defined, fixed and tuned to optimise the trade-off between correctly detecting as many waves as possible and setting the most accurate take-off/end-ride moments.

A post-processing layer of wave characterisation was also implemented to receive each detected wave, refine the definition of the end-ride moment and proceed to the extraction of basic wave-related statistics (e.g., duration and average speed). Refining the end-of-wave limit is an important processing step, due to the range of possibilities of exiting the wave (e.g., kick out, nose dive, and uncontrolled wipe out), which frequently lead to the detection of unexpected wave limits. Each wave period was, therefore, restrained: its last moment should correspond to the last occurrence of a linear acceleration measurement above 90% of the mean in-wave acceleration. The refined wave period was then validated if it lasted more than 3 s, with peaks of linear acceleration and speed in accordance with the defined thresholds.

#### 3.2.2. Stances

Non-wave time-windows were fed to the stance detection block. This layer should identify periods when the surfer is laying vs. sitting on the surfboard, based on the distribution of the acceleration of the gravity component in the sensor frame’s axis. This means that measurements of nearly 9.8 m/s^2^ in *Y*- or *Z*-axis led to the direct classification of sitting or laying stances, respectively.

The same block is responsible for the attribution of the label *other*, a rejection class assigned when none of the criteria that describe a known activity are verified. These moments frequently correspond to transition periods between known activities, and are mostly short, ambiguous or irrelevant; as such, this class is not associated to any ground truth labels, i.e., it was not annotated.

#### 3.2.3. Laying Activities

Several events can occur during laying periods, which are relevant to identify and characterise, namely paddle, sprint paddle, dives, and idle laying (with no substantial movement). The characterisation of such events was separately performed in an individual block, with two different implementations (based on time- or frequency-domain features), compared in the scope of this work.

##### Time-Domain Features

The first implementation corresponds to the paddle detection algorithm detailed in [17], in which, upon finishing a laying event, data would proceed to a next block responsible for determining if paddle occurred in that timeframe. The acceleration of gravity measured in the *X*-axis was considered the most appropriate signal to support this identification. Maximum and minimum peaks were detected in this signal, and their timelapse was measured to perform an estimation of signal periodicity. If the signal was considered sufficiently periodic, a paddle label was assigned.

This version of the laying activity detection block solely uses time-domain features to base its predictions, and is confined to a binary decision (paddling vs. not paddling) whenever data from a new laying period arrive. Moreover, this method assesses the entire laying window, without employing any segmentation technique, at the expense of an expected loss of performance whenever several different activities occurred in the same laying window. This motivated the exploitation of frequency-domain features to solve the problem of laying activities characterisation.

##### Frequency-Domain Features

**Figure 3:**
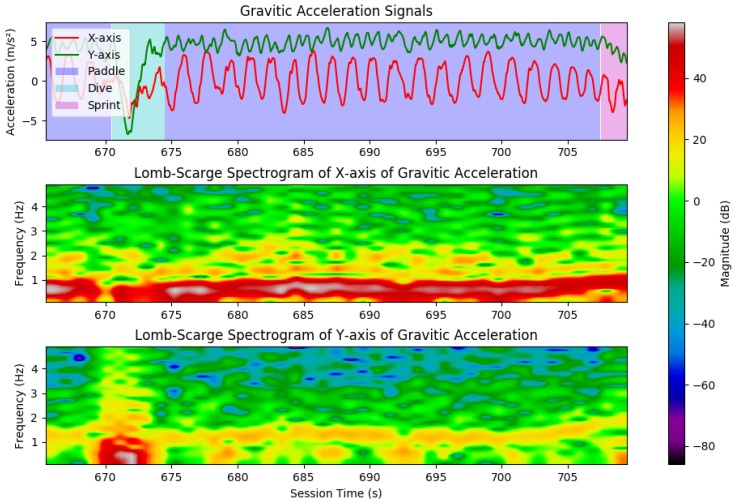
Segment of surf session, evidencing paddle periods and dive moments: (**Top**) the *X* and *Y* components of gravitic acceleration, with background color as the output of the laying events classification block; (**Middle**) the obtained spectrogram for the gravitic acceleration in the *X*-axis; and (**Bottom**) the obtained spectrogram for the gravitic acceleration in the *Y*-axis. Note the high energy content for low frequencies during paddling in the *X*-axis, and the peaks in low frequencies in the *Y*-axis during dives.

As previously stated, by observing the *X*, *Y*, and *Z* gravity acceleration signals derived from sensor fusion, one can verify that these signals, particularly the *X* and *Y* axes, are quite periodic in nature during paddle periods, with an almost sinusoidal behaviour, with distinct regular peaks and valleys (see Figure 3 for examples of paddling periods). The *X*-axis presents the signal with higher magnitudes, whereas the *Y*-axis shows a less intense variation, but with approximately twice the number of peaks and valleys than the *X*-axis signal, when considering the same time frame. For inactive laying periods, these signals tend to be much lower in magnitude, with no detectable periodicity.

Considering the positioning and orientation of the smartphone during data collection, it is likely that this periodicity arises from the paddling movements performed by the surfers, which translate into a steady “wobble” of the torso-surfboard complex, where each valley corresponds to a left arm paddle and each peak to a right arm paddle.

As such, the characteristic periodicity of these signals was exploited to further segment and classify laying periods in one of four classes (paddle, sprint paddle, dives, and laying still), by performing spectrographic analysis. Since the collected IMU signals were not completely evenly time sampled, and to avoid performing linear interpolation for sampling frequency normalisation, Lomb-Scargle periodograms were calculated for successive windows. The Lomb-Scargle periodogram algorithm allows the calculation of frequency content of unevenly time sampled signals [28].

Each of the identified laying periods was subdivided into 2 s windows, with 1 s overlap: since the average paddling frequency was around 0.5 Hz (one arm paddle per second), at least 2 s data were necessary to identify a full paddle period (left and right arm). The Lomb-Scargle periodogram of the *X* and *Y* gravity acceleration signals of each window was calculated to create a spectrogram of these signals during the identified laying period, considering evenly spaced out frequencies from 0 to 5 Hz. The frequency component with highest magnitude was identified for each 2 s window, for both acceleration axes. Each of these 2 s windows was then classified as one of the following:

- Paddle
  Paddle is present when the gravity acceleration component in the *X*-axis has a high energy content in low frequencies (usually under 1 Hz), with the *X*-axis being of higher energy than the *Y*-axis. Examples of paddle periods and corresponding spectrogram can be seen in Figure 3, in dark blue.
- Dives
  Dives can be identified based on their characteristic “dip” in the *Y* component of gravitic acceleration, corresponding to the downward movement of the surfer while diving. In the frequency domain, this translates into a peak in the maximum magnitude of the *Y*-axis signal in low frequencies, larger than the *X*-axis. Several dive moments can be seen in Figure 3, in light blue.
- Sprint Paddle
  If paddle periods were identified, they were further processed to identify sprint paddle, an activity characterised by an increase in paddling frequency and magnitude of linear acceleration in the *Y* and *Z* axes, as a surfer approaches the cusp of a wave. If a paddle window had its highest energy content in a higher frequency, and high enough YZ linear acceleration (that corresponds to the acceleration felt when on a wave’s cusp), it was classified as sprint paddle.
- Laying still
  Any other remaining windows that were not classified as paddle, sprint paddle, or dives, were set by default as regular laying periods, with no associated events or activities.

After the identification of the aforementioned events during a laying period, with each 2 s window being classified as laying, paddle, sprint paddle, or duck dive, these windows were further processed to remove outlier classifications, such as windows that are embedded in periods of one activity and are classified as another. This is performed by comparing each window to its neighbouring windows and determining if it is an isolated instance of a given classification: in these cases, the attributed event to that window is set to correspond to its neighbouring events, thus leading to an overall smoother classification of activities.

## 4. Results

### 4.1. All Events’ Detection Performance

The evaluation of the algorithm’s performance concerning the detection of all surf session events was conducted by taking the *All events dataset* and computing the confusion matrix. To this end, samples were taken in periods of 1 s, and attributed their respective prediction and ground truth labels, derived from the aforementioned thorough annotation process. According to the described methodology, two confusion matrices were computed, each considering a different version of the laying activities profiling algorithm.

Table 2 exhibits the confusion matrix for the five-label output algorithm, solely based on time domain features. Since, in this case, we only aimed to distinguish paddle from still laying, and not dive or sprint paddle, these activities were considered to belong to class paddle for ground truth purposes. In our previous work [17], we presented the confusion matrix of the activities detected based on time-domain features grouped by stance detection, i.e., the paddle activity comprised both paddle and lay activities’ data, since the *All events dataset* was small, with low representativeness of still laying activities. In this work, since the dataset was increased, Table 2 discriminates the results in paddle and lay activities, according to the actual label attribution by the algorithm. It is clear that the number of annotated laying samples is still reduced compared to the number of annotated paddle instances. This aspect is considered in the discussion of the results in the next section. As one can infer from the confusion matrix, an overall accuracy of 87.5% was achieved for the detection of all activities of interest using only features from the time domain.

Table 3 presents the results of the newly proposed algorithm, which bases its characterisation of the laying activities on frequency-domain features. This version of the algorithm predicts more activities than the previous; in total, it considers seven different output labels, four of which consisting of activities that occur while the surfer is laying on the surfboard. Overall, in this case, an accuracy of 88.1% was attained for the classification of all activities.

### 4.2. Wave Detection Performance

Wave riding is the most important event in the context of a surf session. As such, wave detection performance was separately studied from the remaining events, with as much evidence as possible, considering all acquired data of the *Waves dataset*. Table 4 presents the results of this validation using two metrics: precision, which weights the amount of correct detected waves in the entire universe of detected waves, and recall, a measure of the amount of annotated waves that were indeed detected.

The cases when the algorithm detected a wave during a period in which the surfer is not visible in the ground truth videos were also quantified, as presented in the last column of Table 4. These events were not considered for computation of the performance metrics.

## 5. Discussion

Direct comparison of the five-label output event detection algorithm with the preliminary results reported in [17] demands the recomputation of the confusion matrix presented in Table 2 to solely feature a total of four labels instead of five, by fusing paddle and lay instances. From this rearrangement, an overall event detection accuracy of 93% was achieved using the *All events dataset* described in Table 1. This value is just slightly inferior to that reported in [17]—95%, computed from the analysis of only three surf sessions—which proves the coherence and robustness of the algorithm itself. However, in this work, the confusion matrix presented in Table 2 discriminates paddle from still lay detections, in accordance with the labels returned by this version of the algorithm (see Figure 2). When considering this extra activity, the performance in terms of accuracy drops to 87.5%, due to the frequent confusion of still laying with paddle. This problem can relate with the difficulty to annotate lay samples using video ground truth (e.g., situations when the surfer is too far away), but we believe it is mostly inherent to the nature of the algorithm itself: since there are no segmentation operations implemented over the laying periods arriving to the laying activity detection block, each period is simply assigned a single label, even if it featured more than one activity. This means that a sequence such as *paddle–lay–paddle* would be simply labelled as paddle, if the periodicity threshold was surpassed, or lay, if it was not. This fact affects lay detection performance in particular, because lay events are scarce (as the low number of annotated lay samples in Table 2 indicates) and frequently occur between paddling periods, which means that the surfer’s stance does not change, and, thus, the entire period will be assigned a single label—usually paddle because it was the most frequent activity in such periods. This problem revealed the importance of further segmenting laying periods.

Thus, the new version of the laying activities detection block started by implementing a segmentation method, splitting the laying period. Then, frequency-domain features were extracted and used to distinguish four types of laying activities. This flow of operations was expected to neutralise the problem identified and discussed in the previous paragraph, while increasing overall activity classification robustness. An accuracy of 88.1% was finally attained for the detection of all seven events. This means that a slightly superior performance was achieved for the classification of a higher number of activities, leading to a better description of the entire surf session. Laying activities are still the hardest to distinguish, with lay and dive instances frequently confused with paddle by the algorithm. The impact of this situation is, however, diminished if we take into consideration the low number of annotated lay and dive instances, which constitute a total of roughly 23 min (only 5% of all annotated data). The most frequent confusion occurs between sprint paddle and waves, which is expected due to the ambiguity of the annotation process in defining the boundaries of these activities. The algorithm was also optimised not to miss any wave data, as described in [17], thus the inclusion of the latest moments of sprint paddle in wave periods segmentation was likely by design. Therefore, despite the higher complexity and computational load associated to the extraction of frequency-domain features (compared with the time-domain approach), these were deemed more suitable to describe and model the problem of laying activities’ distinction.

Regarding wave detection performance alone, one can highlight the achievement of 90.3% accuracy of wave detection with second precision, considering the annotated times as ground truth. Then, due to the importance of not missing any waves while still minimising false detections, the whole universe of wave events (327) was taken to compute precision and recall of wave detections, with values of 97.47% and 94.19%, respectively. This outcome was also coherent with the preliminary results reported in [17] based on a much smaller dataset (in this work, the number of annotated waves increased to more than double), reporting the same precision and just slightly inferior recall.

These results shall stand as evidence of the appropriate performance and robustness of the algorithm. Moreover, it is important to highlight its user-independent nature, the span of conditions under which data collections took place (namely, different sea and weather conditions), and its near real-time event classification, which simultaneously increased the complexity of this work while making its outcome more appealing for usage in a real-world scenario. Besides our previous work [17], no other studies were found for comparison when it comes to the classification of all events in a surf session.

### Challenges and Limitations of the Study

This study presented some challenges and limitations which are worthy of a thorough discussion, mostly concerning the data collection setup and data annotation procedure, both associated to constraints of the surf activity itself. As such, the limitations of this study include the low percentage of annotated data of some activities of interest, allied with an ambiguous annotation process in some cases.

Video annotation was constrained by several difficulties, namely: (1) lack of direct sight of the surfers at all times; (2) impossibility of determining with certainty the identity and/or on-going activity of surfers standing too far away from the shore; and (3) ambiguous determination of ground truth start and end times of some surfing events (e.g., exact moment when the surfer initiates a dive or the moment when regular paddle becomes sprint paddle). Nonetheless, 44% of the data was annotated—approximately 7.5 h—which was considered sufficient to successfully validate activity classification.

The introduction of techniques that consider the sequential dependency between activities in a surf session could be an appropriate next step to improve the performance of event detection. Moreover, the addition of more cameras (another camera at a different location on the beach and/or a drone for top view of the surfers) could assist the problem of data annotation.

In fact, a drone was used in some data collections; however, its utilisation also has some limitations: (1) it is dependent on weather conditions, namely strong wind, making it impossible to use in every scheduled data collection; (2) each flight only lasts 15–20 min due to the battery life of the device, i.e., we cannot record the entire session with a single drone (even if we switch the batteries and send it back, several minutes are lost); (3) several sessions took place with two surfers at once, whom often cannot be spotted at the same time by the drone, i.e., we would not have ground truth data for both surfers under analysis during most of the time of each flight. While it could be interesting to use both beach video camera and drone images for annotation, the amount of data we would be able to annotate based on drone images would be very little compared to the total amount of acquired data that we can annotate with the beach video camera. Furthermore, it would imply the creation of different annotation protocols within the same experiment, with different synchronisation processes, which could be another source of error for little gain. Therefore, we opted for leaving all drone images out of these experiments, and plan to use them later on in further developments of our work, namely to study the surfers’ in-wave movements with great detail.

## 6. Conclusions

This manuscript conveys an extension of the work in [17] by bringing forward an extended validation of the event detection algorithm with an enlarged and more mature dataset, and a new method that improved the characterisation of laying events. The algorithm proved to be 88.1% accurate in distinguishing seven surf-specific activities—wave riding, paddling, sprint paddling, diving, laying, and sitting on the board—with second precision. Besides its promising results, our overall solution is also user-independent and has a nearly real-time response, which reiterates its maturity and market value.

As future work, the proposed solution will be integrated and validated in real-time and real-world scenarios to ensure its reproducibility. Moreover, a manoeuvre detection block shall be implemented, aiming to characterise and evaluate in-wave events, and the potential of creating a model to assess the correctness of the movements of each surfer will be explored. Different segmentation techniques shall also be explored to assess their impact in the performance of our system. Finally, we shall explore the use of top view images (collected using a drone) and/or a second camera fixed at the beach to improve the process of data annotation, and perform a more thorough validation, not only of the activity recognition module, but also of further extraction of metrics of surfing performance (e.g., rotation angles).

## Figures and Tables

**Table 1:** Datasets description.

	Waves Dataset	All Events Dataset
**No. sessions**	17	13
**No. users**	13	9
**Average session duration**	1h18	1h19
**% Annotated session time**	5%	44%
**No. waves**	327	269
**Average wave duration**	8.97 s	8.67 s

**Table 2:** Confusion matrix (%) for the detection of all events based on time-domain features alone. Rows: true labels; Columns: predicted labels.

	Paddle	Wave	Sit/Wait	Lay	Other	No. Annotated Seconds *
**Paddle**	**87.94**	0.45	0.65	5.15	5.81	12,867
**Wave**	1.25	**89.23**	1.03	0.22	8.27	2238
**Sit/wait**	2.06	0.00	**94.57**	0.65	2.72	10,924
**Lay**	91.09	2.01	1.72	**3.74**	1.44	1044

* Percentage of annotated data: 44%.

**Table 3:** Confusion matrix (%) for the detection of all events based on time-domain (waves and stances) and frequency-domain (laying activities) features. Rows: true labels; Columns: predicted labels.

	Paddle	S. Paddle	Wave	Sit/Wait	Lay	Dive	Other	No. Annotated Seconds *
**Paddle**	**85.56**	1.19	0.11	0.70	4.53	2.73	5.18	12,272
**S. Paddle**	10.84	**41.77**	26.10	0.00	8.43	2.01	10.84	249
**Wave**	0.31	0.04	**90.30**	0.94	0.27	0.45	7.69	2238
**Sit/wait**	0.58	0.00	0.00	**94.98**	1.31	0.82	2.31	10,924
**Lay**	21.65	0.10	2.01	1.72	**63.98**	8.24	2.30	1044
**Dive**	21.68	1.73	0.87	0.29	8.67	**49.71**	17.05	346

* Percentage of annotated dataset: 44%.

**Table 4:** Wave detection performance overview.

Total No. Waves	Precision	Recall	% Detected Waves Not Caught on Video
327	97.47%	94.19%	4.43%
